# Cdk5 phosphorylates non-genotoxically overexpressed p53 following inhibition of PP2A to induce cell cycle arrest/apoptosis and inhibits tumor progression

**DOI:** 10.1186/1476-4598-9-204

**Published:** 2010-07-31

**Authors:** Amrendra K Ajay, Ankur K Upadhyay, Sandeep Singh, Maleppillil V Vijayakumar, Ratna Kumari, Vimal Pandey, Ramanamurthy Boppana, Manoj K Bhat

**Affiliations:** 1 National Centre for Cell Science, NCCS Complex, Ganeshkhind, Pune - 411007, India; 2 Current Address: Ranbaxy Research Laboratory, Gurgaon, Haryana, India; 3 Current Address: H. Lee Moffitt Cancer Center, Tampa, Florida, USA

## Abstract

**Background:**

p53 is the most studied tumor suppressor and its overexpression may or may not cause cell death depending upon the genetic background of the cells. p53 is degraded by human papillomavirus (HPV) E6 protein in cervical carcinoma. Several stress activated kinases are known to phosphorylate p53 and, among them cyclin dependent kinase 5 (Cdk5) is one of the kinase studied in neuronal cell system. Recently, the involvement of Cdk5 in phosphorylating p53 has been shown in certain cancer types. Phosphorylation at specific serine residues in p53 is essential for it to cause cell growth inhibition. Activation of p53 under non stress conditions is poorly understood. Therefore, the activation of p53 and detection of upstream kinases that phosphorylate non-genotoxically overexpressed p53 will be of therapeutic importance for cancer treatment.

**Results:**

To determine the non-genotoxic effect of p53; Tet-On system was utilized and p53 inducible HPV-positive HeLa cells were developed. p53 overexpression in HPV-positive cells did not induce cell cycle arrest or apoptosis. However, we demonstrate that overexpressed p53 can be activated to upregulate p21 and Bax which causes G2 arrest and apoptosis, by inhibiting protein phosphatase 2A. Additionally, we report that the upstream kinase cyclin dependent kinase 5 interacts with p53 to phosphorylate it at Serine20 and Serine46 residues thereby promoting its recruitment on p21 and bax promoters. Upregulation and translocation of Bax causes apoptosis through intrinsic mitochondrial pathway. Interestingly, overexpressed activated p53 specifically inhibits cell-growth and causes regression *in vivo *tumor growth as well.

**Conclusion:**

Present study details the mechanism of activation of p53 and puts forth the possibility of p53 gene therapy to work in HPV positive cervical carcinoma.

## Background

p53, a major tumor suppressor or guardian of the genome is mutated, deleted or inactivated in various cancers [1-4]. Almost all human papillomavirus (HPV) infected cancer cells contain wild-type p53. p53 is non-functional as HPVE6 protein abrogates its function either by ubiquitin-dependent and independent degradation [5], by inhibition of acetylation or by repressing p53-dependent downstream molecular pathways [6]. Though, E6 associates with p53 for its degradation [4]; there are contradictory reports on the inhibition and activation of p53 pathways by E6 [7,8].

Ectopic expression of p53 in cancer cells lacking p53 or harboring mutant and/or abrogated wild-type p53, have contrasting effects on cell-fate. In p53 null cancer cells, p53 overexpression causes cell cycle arrest and apoptosis [9]. However, in virus infected cells harboring wild-type p53, overexpression of p53 does not induce cell cycle arrest and apoptosis [10]. Till date there are only three reports describing the consequences of p53 overexpression in HPV-positive cells and results obtained leave ample scope for debate [10-12]. Disparity among these reports may be due to differences in adenoviral multiplicity of infection. Taken together, the role of p53 overexpression in HPV-positive cells remains obscure. In HPV-positive cells, E6 works at different hierarchal levels in p53 pathway. It degrades p53, p21 and Bax causing impairment in cell cycle arrest/apoptosis [13,14] and making p53 activation more difficult.

With recent developments in efficient gene delivery systems and the prospect of gene therapy making a come-back [15] it's likely that p53 based therapy may become a reality [2]. p53 executes its tumor suppressor activity by triggering cell cycle arrest and apoptosis. However, the factors that facilitate selection between cell cycle arrest and/or apoptosis are not well-understood. It has been reported that p21 is most important transcriptional targets of p53 for causing cell cycle arrest [16] and p53 executes apoptosis through Bax transcription [17]. To study the role of p53 in E6-positive cells, we developed a novel isogenic HeLa cells with Tet-On-regulated p53 expression. Tet-On system exhibits tight-on/off regulation and is devoid of pleiotropic effects. Moreover, rapidly high induction levels are achievable and the inducer, doxycycline (Dox), is well-characterized.

p53 overexpression does not promote cell cycle arrest and apoptosis in HeLa cells. We demonstrate that protein phosphatase 2A (PP2A) controls p53 functions and its inhibition activates p53, causing cell cycle arrest/apoptosis *in vitro *and tumor growth inhibition *in vivo*. Interestingly, cyclin dependent kinase 5 (Cdk5) regulates p53 phosphorylation essential for its activation. Taken together, we propose that non-genotoxically overexpressed p53 can be activated by inhibiting its dephosphorylation in HPV-positive cervical cancer cells. This strategy may be of therapeutic importance in p53 associated gene therapy [18-20].

## Materials and methods

### Chemicals and cell-lines

Antibodies against p53 (DO-1 and FL-393), GFP, pCdk5, Cdk5, p35, Bcl-2, cytochrome-C, PARP, COX-IV, β-Tubulin, β-Actin, HRP-linked secondary antibodies, Control, p53 and PP2A siRNAs were purchased from Santa Cruz Biotechnology (Santa Cruz, CA). Phospho-specific p53 antibodies were purchased from Cell Signalling Technology (Danvers, MA). FITC and rhodamine-conjugated secondary antibodies were purchased from KPL (Gaithersburg, MD). APO-Direct TUNEL kit, Matrigel and Bax antibody was purchased from BD (Franklin Lakes, NJ). Dox was purchased from Sigma (St. Louis, MO). Hygromycin B and Tet system approved serum was purchased from Clontech (Mountain View, CA). G418 and MTT were purchased from USB (Cleveland, OH). Okadaic acid (OA), mitotracker dye, DMEM, FBS and Lipofectamine2000 were purchased from Invitrogen (Carlsbad, CA). Cdk2/5 inhibitor, PFTα and U0126 were purchased from Calbiochem (Gibbstown, NJ). Cdk5 siRNA was purchased from Dharmacon Inc (Lafayette, CO). Development of cell-lines is described in additional file 1.

### Plasmids and transfection

pC53-SN3 and pG13CAT were a kind gift from Dr. Bert Vogelstein, John Hopkins, Baltimore, MD. p53 fragment of pC53-SN3 was sub cloned in *BamH1 *site of pTRE and renamed as pTREp53. pG13CAT contains 13 repeats of p53 consensus binding site inserted in the 5' end to polyomavirus basal promoter linked to CAT reporter gene. Cells were co-transfected with 2 μg of pG13CAT and 0.5 μg of pEGFPC1 which serves as an internal control for transfection. Bcl-2 fragment from pRc/CMVBcl-2 (kind gift from Dr. S. Soddu, Regina Elena Cancer Institute, Italy) was excised by *HindIII *and cloned into pTRE2 to obtain pTRE2Bcl-2. Cells were transfected with either 2.0 μg (for 35 mm plate) or 0.5 μg (for 96 well plate) plasmid by Lipofectamine2000 transfection reagent as per manufacturer's instructions.

### Clonogenic-survival assay

Cells (500) were treated with indicated concentrations of Dox, OA or Cdk2/5 inhibitor based on the experimental design and incubated for 48 h. Cells were further grown for 21 days and thereafter colonies on the plate were stained with crystal-voilet.

### Electrophoretic mobility shift assay (EMSA)

To visualize the DNA-binding activity of p53 in nuclear extracts of HTet23p53, HTet26p53, HTet43GFP and HeLa cells, EMSA was performed. After treatment with Dox, cells were harvested for the preparation of cytoplasmic and nuclear fractions by using nuclear extraction kit as per manufacturer's instructions (Chemicon, Billerica, MA). Nuclear lysates were incubated for 45 min at 4°C and cleared by centrifugation at 15,000 ×g for 15 min at 4°C. Equal amount of nuclear proteins were used for the binding reaction. Complementary oligonucleotides containing the sequences corresponding to putative p53 binding site (forward, 5'-GAACATGTCTAAGCATGCTG-3'; reverse, 5'-CAGCATTCTTAGACATGTTC-3') were annealed and 5'-end-labeled with 2 micro curie (ΔCi) [γ-^32^P] ATP using 10 U of T4 polynucleotide kinase (Invitrogen) for 90 min. Binding reaction was carried out in a final volume of 20 μl consisting of 10 mM Tris.HCl (pH 7.5), 50 mM NaCl, 1 mM DTT, 1 mM EDTA, 2.5% glycerol, 1 μg deoxyinosinic deoxycytidylic acid [poly(dI-dC)], 300 ng BSA, 5 μg nuclear extract, and 2 μl of [γ-32P] labeled oligonucleotide probe. Reaction mixtures were incubated for 20 min at room temperature. Samples were resolved on a native polyacrylamide gel. Gel was dried under vacuum at 80°C for 45 min by gel dryer (Bio-Rad) and DNA-protein complex were visualized by autoradiography.

### Chloramphenicol acetyl transferase assay

Cells were co-transfected with pG13CAT and pEGFPC1 expression vector using Lipofectamine2000 as described in transfection section. After 18 h post-transfection, p53 was induced with Dox for 48 h with or without PFTα pretreatment for 1 h. CAT assay was performed as described earlier [2] except that the reaction time was reduced to 30 min at 37°C. Spots were quantified by phosphoimager (Bio-Rad). GFP intensity was directly measured from the cell lysates to check or correct for equal transfection efficiency as well to normalize the reporter activity. The fluorescence intensity of GFP in equal amount of lysate was measured by fluorimeter (Fluoroskan Ascent FL, Fisher Scientific) with excitation at 485 nm and emission at 510 nm.

### SiRNA transfections

Cells were transfected with 100 nM control or p53 siRNA using Lipofectamine2000 [21]. Eighteen hour post-transfection, Dox was added with or without OA and further incubated for 48 h. Thereafter, western blot or MTT assay was performed. To knock-down PP2A and Cdk5; Cdk5 siRNA was transfected 12 h prior to PP2A siRNA transfection and then incubated with Dox for 48 h.

### Immunoprecipitation and Chromatin-immunoprecipitation (ChIP) assay

After indicated treatment cells were lysed in RIPA buffer. Equal amount of protein (400 μg) was taken and lysates were pre-cleared with 50 μl protein A/G-plus agarose for 30 min. Fifty microgram lysates were run as input. Agarose beads were pelleted and supernatant was incubated with p53 specific antibody overnight at 4°C. Fifty microliter protein A/G-plus agarose was added in antibody-antigen complex with gentle shaking for 4 h at 4°C. The protein A/G-plus was separated by centrifugation at 4,000 rpm. Target and its associated proteins were disrupted and resolved on SDS-PAGE. The expression of Cdk5 and p53 was detected by western blotting.

For chromatin-immunoprecipitation assay cells or homogenized tumors which were earlier fixed with 1% para-formaldehyde for 15 min, were lysed with 500 μl of lysis buffer [5 mM PIPES (pH 8.0), 85 mM KCl and 1% NP-40]. After centrifugation (5000 rpm), nuclear pellets were resuspended in 150 μl buffer [50 mM Tris-Cl (pH 8.0), 10 mM EDTA and 1% NP-40]. To fragment DNA to approximately 500 bps, samples were sonicated and centrifuged for 10 min. Samples were diluted 10-fold in IP buffer [16.7 mM Tris-Cl (pH 8.0), 167 mM NaCl, 1% NP-40 and 1 mM EDTA]. Samples (400 μg) were incubated with anti-p53 or anti-goat IgG overnight. Remaining solutions (10-times diluted) were used as input. Protein A/G-plus agarose beads pre-blocked with salmon-sperm DNA were added to antibody-antigen complexes and incubated for 4 h. Immune-complexes were centrifuged and washed with buffer [20 mM Tris-Cl (pH 8.0), 150 mM NaCl, 0.5% SDS, 1% Triton X-100 and 2 mM EDTA] twice and with buffer containing 250 mM NaCl. Immune-complexes were eluted by 50 μl of buffer [1% SDS and 0.1 M NaHCO_3_] twice. Then 20 μl of 5 M NaCl was added and incubated at 65°C overnight. DNA was precipitated with ethanol. RT-PCR was performed with promoter primer pairs for p21 (F) 5'-GGC TGG TGG CTA TTT TGT CC-3', (R) 5'-TCC CCT TCC TCC CTG AAA AC-3', and bax (F) 5'-AGC GTT CCC CTA GCC TCT TT-3' and (R) 5'-GCT GGG CCT GTA TCC TAC ATT CT-3' at annealing temperature 57°C and 59°C respectively.

### Mitochondrial and cytosolic fractionation

HTet26p53 cells were swelled in ice-cold hypotonic HEPES buffer [10 mM HEPES (pH 7.4) 5 mM MgCl_2_, 40 mM KCl, 1 mM PMSF and protease inhibitor cocktail] for 30 min and centrifuged at 1500 rpm to pellet the nuclei. The resulting supernatant was centrifuged at 10,000 rpm to pellet mitochondrial fraction. Supernatant was used as cytosolic fraction and mitochondrial pellet was washed with PBS twice. This pellet was lysed in mitochondrial buffer [10 mM MOPS (pH 7.4), 1 mM EDTA, and 4 mM KH_2_PO4, 1% NP-40, protease inhibitor cocktail] and centrifuged at 12,000 rpm for 30 min.

### Immunostaining

Cells grown on Labtek chamber slides were treated with Dox for 48 h and processed for immunofluorescene study as described earlier [21]. Primary antibody against p53 (1:50) was added and incubated for 2 h at room temperature. Following incubation, cells were washed 5 times. Fluorescein isothiocyanate (FITC) or Rhodamine conjugated secondary antibodies (1:100) were added and incubated for 1 h at room temperature. After five washes, vectashield mounting medium containing DAPI was added and slides were examined by a confocal microscope (LSM510, Carl Zeiss, Germany). For mitotracker deep red staining, after indicated treatments cells were incubated with 200 μM of mitotracker dye for 20 min. These were then fixed and processed for immunofluorescence study by incubating with a Bax specific primary antibody and FITC conjugated secondary antibody. Slides were mounted with DAPI containing medium and images were acquired in confocal microscope. Terminal deoxynucleotidyltransferase dUTP nick end labeling (TUNEL) staining was performed as per manufacturer's protocol (BD) except the reaction time was increased to 3 h at room temperature. Cells were washed twice with binding buffer and PI solution was added. Slides were washed, mounted and observed under confocal microscope (META, Carl Zeiss).

### Tumor growth

HTet23p53 or HTet43GFP cells (5 × 10^6^) in 100 μl PBS mixed with 100 μl matrigel were injected s.c. into 4-6 week-old female NOD/SCID mice (Jackson Laboratories). Total 12 mice were injected with HTet23p53 cells on the right flank and 4 mice were injected with HTet43GFP cells on both the flanks. Out of two groups, one was fed on 500 ng/ml Dox in drinking water. Tumor development was monitored. After tumor-size reached to 5-10 mm in diameter, OA (40 pg/mice) was administered at the tumor site. Tumor-sizes were measured weekly by digital Vernier Caliper (Sigma) and tumor volume was calculated by formula V = [1/2 × (large diameter) × (small diameter)^2^.

### MTT assay, FACS analysis and western blotting

For methylthiazole tetrazolium (MTT) assay, 7,500 cells were treated with Dox, OA and/or Cdk2/5 inhibitor as per experimental requirement and assayed for cell survival. For western blotting following indicated treatments, cells were washed thrice with ice-cold phosphate buffered saline (PBS) and lysed in ice-cold lysis buffer (50 mM Tris-Cl, pH 7.5, with 120 mM NaCl, 10 mM NaF, 10 mM sodium pyrophosphate, 2 mM EDTA, 1 mM Na_3_VO_4_, 1 mM PMSF, 1% NP-40 and protease inhibitor cocktail (Roche Diagnostics, Penzberg, Germany). Equal amount of protein was resolved on a polyacrylamide gel. Where ever possible blots were stripped by incubating the membranes at 50°C for 30 min in stripping buffer (62.5 mM Tris-Cl pH 6.7, 100 mM mercaptoethanol, 2% SDS) with intermittent shaking. Membranes were washed thoroughly with TBS and reprobed with required antibodies. Otherwise gels run in duplicates were probed for the desired proteins by western blotting and then compiled.

For FACS analysis cells were plated at a density of 5 × 10^5 ^cells in 35 mm plates and allowed to adhere for 24 h. Cells treated as per experimental requirement were harvested by trypsinization and processed for flow cytometric analysis. The fluorescence of propidium iodide (PI) was measured through a 585 nm filter in a flowcytometer (FACS Calibur, BD) for 10,000 cells. Data were analyzed using cell quest software (BD). Details of these are as published earlier [21,22].

### TUNEL staining

To detect apoptotic cells APO-DIRECT TUNEL assay kit (BD) was used followed by flow cytometric analysis as per the manufacturer's instructions with some modifications. Cells were incubated in DNA-labeling solution for 2 h at 37°C and analyzed by FACS Calibur (BD). PI stains total DNA and FITC conjugated dUTP stains apoptotic cells.

### Reverse-Transcription-PCR

Total RNA from the cells or tumor samples was extracted using TRIzol™ reagent and PCR was performed as described [21] with following primers; p53 (F) 5-CTG AGG TTG GCT CTG ACT GTA CCA CCA TCC-3', (R) 5'-CTC ATT CAG CTC TCG GAA CAT CTC GAA GC-3'; e6 (F) 5'-TGT GTA TGG AGA CAC ATT GG-3', (R) 5'-ATA GTG CCC AGC TAT GTT GT-3'; β-actin (F) 5'-ATC TGG CAC CAC ACC TTC TAC AAT GAG CTG CG-3', (R) CGT CAT ACT CCT GCT TGC TGA TCC ACA TCT GC-3', at annealing temperature of 55°C and p21 (F) 5'-GGC GTT TGG AGT GGT AGA AA-3' (R) 5'-GAC ACC ACT GGA GGG TGA CT-3' at annealing temperature of 59°C for 25-30 cycles.

### Statistical analysis

Statistical comparisons are made using student's paired *t*-test using SPSS10.0 (SPSS Inc., IL) and P*-*value < 0.05 was considered significant.

## Results

### Development and screening of HeLaTet-On p53 inducible cell-system

Seven out of 24 p53 transfected clones (HeLaTet-On-p53 21 to 44) and nine out of 12 GFP transfected clones (HeLaTet-On-pBIEGFP 41 to 52) exhibited induction in the presence of Dox (see Additional file 2A and 2B). Two clones HeLaTet-On-p53-23 S and HeLaTet-On-p53-26 S (represented as HTet23p53 and HTet26p53) along with HeLaTet-On-BIEGFP-43 (represented as HTet43GFP) with low-leaky and high regulatory expression were selected for further studies. Growth properties of clones for 6 days were similar to parental HeLa cells (see Additional file 3A). Also, protein concentration did not alter between the clones and the parental cells (see Additional file 3B). Dox upto 2000 ng/ml was non-toxic (see Additional file 3C).

**Figure 1:**
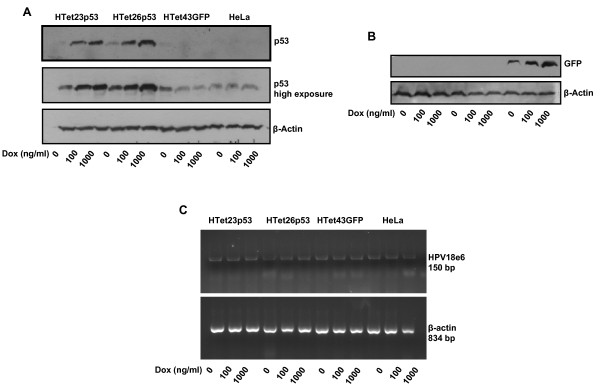
**p53/GFP are tightly regulated by Dox and it does not alter HPV18E6, a viral cell cycle deregulator**. (A) Selected clones (HTet23p53, HTet26p53 and HTet43GFP from Additional file 2) were treated with 100 and 1000 ng/ml of Dox and after 48 h cell lysates were processed by western blotting for p53. β-Actin was used as a loading control. (B) Cells were treated as mentioned in (A) and processed for western blotting to detect GFP. β-Actin was used as a loading control. (C) HPV18 E6 mRNA levels were determined by semi-quantitative PCR. Cells treated with Dox were processed for RT-PCR. β-actin was used as a loading control. HTet43GFP and HeLa cells served as experimental controls.

Tight-regulation of p53 expression was confirmed by addition of 100 and 1000 ng/ml of Dox. p53 expression was induced in response to Dox in a dose-dependent manner (Figure 1A). Also, GFP protein expression was tightly-regulated (Figure 1B). As E6 downregulation induces cell-death, E6 mRNA levels in p53 and GFP expressing clones as well as in parental HeLa cells was detected by RT-PCR. No alteration in e6 expression following treatment with Dox was observed (Figure 1C). p53 localization and nuclear retention is essential for execution of its transcriptional and tumor suppressor activities. However, in cancer cells wild-type p53 is sequestered in cytoplasm by various molecules which prevent its functioning [23]. p53 induced in response to Dox in a dose dependent manner is predominantly localized in the nucleus (green represents p53 staining and blue represents DAPI for DNA stain in the nucleus) (see Additional file 4A and 4B). No alteration in p53 protein expression was detected in Dox treated HTet43GFP (red-staining) and parental HeLa cells (green-staining) (see Additional file 4C and 4D). In HTet43GFP cells GFP protein expression (green-staining) is tightly-regulated by Dox (see Additional file 4C).

### p53 overexpression does not cause cell cycle arrest or growth inhibition in HeLa cells even though it possesses DNA binding activity

**Figure 2:**
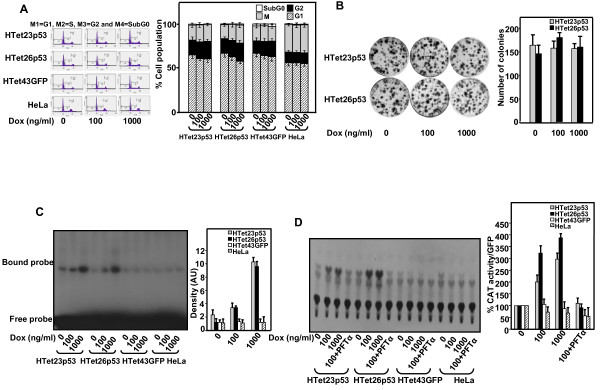
**Overexpressed p53 neither causes cell cycle arrest nor inhibits growth even though it exhibits *in vitro *as well as *in vivo *DNA binding**. (A) Cells treated with 100 and 1000 ng/ml of Dox were incubated for 48 h and processed for flow cytometric analysis by PI staining. Graphical representation of FACS data, bar graph represents % cell populations in each cell cycle phase (± S.E.). (B) Five hundred cells seeded in 35 mm plates and treated with Dox as mentioned in (A) were allowed to grow for 21 days with replacing Dox containing medium every 4 day. Cells in plates were stained with crystal violet stain and colonies were counted. Bar graph represents average of colony number per plate (± S.E.) from three independent experiments. (C) For *in vitro *DNA binding, after 100 and 1000 ng/ml Dox treatment for 48 h, nuclear extracts derived from HTet23p53, HTet26p53, HTet43GFP and HeLa cells were incubated with a γ-^32^P-labeled DNA probe having the consensus p53 binding motif. Bar graph represents densitometric values of autoradiograph (± S.E.). (D) *In vivo *transactivity of p53 was determined by transfecting pG13CAT as well as pEGFPC1 plasmid in HTet23p53, HTet26p53, HTet43GFP and HeLa cells following Dox treatment of 48 h. Percentage CAT activity was calculated by measuring the acetylations of ^14^C-chloramphenicol on thin layer chromatography using phosphorimager. Percent CAT/GFP for HTet43GFP was calculated by dividing with GFP reading for without Dox treated cells only. PFTα was used for the specificity of the p53 activity. Bar graph represents %CAT values normalized to GFP fluorescence for transfection efficiencies from three independent experiments (± S.E.).

PI staining for the cell cycle analysis depicted no alteration in cell cycle phases in p53 overexpressing cells as compared to HTet43GFP or HeLa cells (Figure 2A). Long-term consequence of p53 overexpression was investigated by clonogenic-survival assay. Almost equal numbered and sized colonies were formed by p53 overexpressing HTet23p53 and HTet26p53 cells (Figure 2B). As Dox-induced p53 was localized in the nucleus, its *in vitro *DNA-binding activity by electrophoretic mobility shift assay (EMSA) and *in vivo *transcriptional activity by chloramphenicol acetyl transferase (CAT) reporter gene was evaluated. Increased binding of p53 to its consensus sequence in HTet23p53 and HTet26p53 but not in HTet43GFP and HeLa cells after Dox addition was detected (Figure 2C). Also, there was increase in CAT activity in p53 overexpressing HTet23p53 and HTet26p53 cells and no increase was detected in HTet43GFP and HeLa cells (Figure 2D). Specificity of CAT activity was confirmed by PFTα treatment.

### Activation of p53 by inhibition of phosphatase

**Figure 3:**
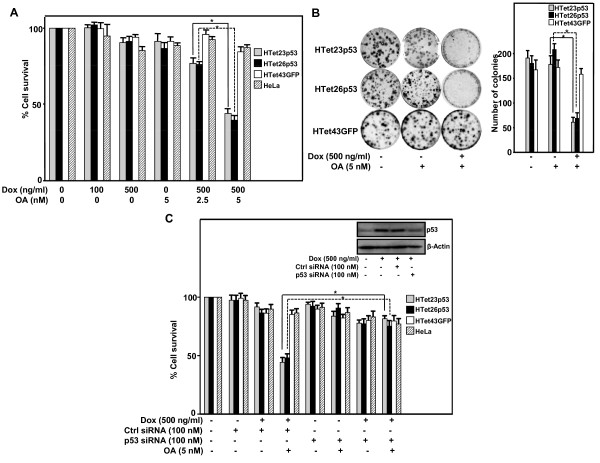
**Inhibition of PP2A causes cell-growth inhibition in a p53 dependent manner**. (A) HTet23p53, HTet26p53, HTet43GFP and HeLa cells were treated with indicated concentrations of OA with or without Dox for 48 h. Thereafter MTT assay was performed. Bar represents variation within the wells of an experiment (± S.E.). *Represents P < 0.01. (B) Five hundred cells plated were treated as mentioned in (A). After 48 h cells were washed and incubated for 21 days. Cells were stained with crystal-violet and colonies were counted. Bar graph represents average colony number per plate (± S.E.) from three experiments. *Represents P < 0.01. (C) HTet23p53, HTet26p53, HTet43GFP and HeLa cells were transfected with control or p53 siRNA in 96 well-plates, 18 h post-transfection cells were treated with Dox and/or OA and MTT assay was performed after 48 h. Bar graph represents variation within the wells of an experiment (± S.E.). *Represents P < 0.05. HTet26p53 cells were transfected with control or p53 siRNA. Eighteen hour post-transfection cells were treated with Dox and further incubated for 48 h. MTT for cell survival evaluation or western blot analysis detection of p53 was performed.

To inhibit the phosphatase, okadaic acid (OA), a potent and specific inhibitor of PP1A and PP2A was utilized. Inhibitory-effect of OA for PP1A and PP2A is concentration-dependent. Inhibitory-concentration (IC_50_) for PP2A is 0.1-10 nM and for PP1A it is 50-1000 nM [24]. We used 5 nM OA to specifically inhibit PP2A and this concentration inhibits almost 100% of phosphatase activity in the cells [25]. Dose-dependent growth inhibition (2.5 nM OA caused 25% whereas 5 nM caused 60%) was observed in p53 overexpressing HTet23p53 and HTet26p53 as compared to HTet43GFP and HeLa cells (Figure 1A). OA alone did not significantly affect cell-survival (Figure 3A). Decrease in colony number and size in p53 overexpressing OA treated cells (HTet23p53 and HTet26p53) was observed as compared to HTet43GFP cells (Figure 3B). To confirm that indeed p53 specifically inhibits cell-growth in the presence of OA, p53 siRNA was transfected which decreased the levels of overexpressed p53 as compared to transfection with Ctrl siRNA (Figure 3C inset). Silencing p53 reduces cell-death by 30-35% in p53 overexpressing cells as compared to HTet43GFP cells or Ctrl siRNA transfected cells (Figure 3C).

### Activated p53 executes its anti-proliferative action through cell cycle arrest and apoptosis by specific promoter recruitment

**Figure 4:**
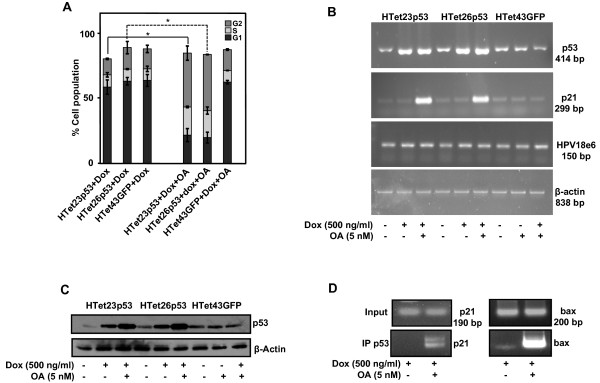
**p53 executes cell-growth inhibition by cell cycle arrest and apoptosis**. (A) HTet23p53, HTet26p53 and HTet43GFP cells treated with Dox with or without OA and were processed for cell cycle analysis. Bar graph represents average of three independent experiments (± S.E.). *Represents P < 0.05. (B) Cells treated as mentioned in (A) were processed for RT-PCR by utilizing p53, p21, HPV18e6 and β-actin primers. (C) HTet23p53, HTet26p53 and HTet43GFP cells were treated with Dox with or without OA for 48 h and western blot for p53 was performed. β-Actin served as loading control. (D) ChIP assay demonstrates *in vivo *interaction of overexpressed p53 with the p21 and bax promoters. Cells were treated with Dox with or without OA for 48 h and processed for ChIP assay.

To evaluate whether growth-inhibition is caused by cell cycle arrest or apoptosis, cell cycle analysis was performed. Approximately 10% increase in S phase and 30% increase in G2 phase in p53 overexpressing cells as compared to GFP expressing cells was observed (Figure 4A). p21, a p53 transcriptional target is a dominant effecter molecule that causes cell cycle arrest. Its transcript level increased significantly following OA treatment in p53 overexpressing HTet23p53 and HTet26p53 cells as compared to GFP expressing cells. Under similar experimental conditions no change in HPV18E6 mRNA was observed (Figure 4B). TUNEL assay using FACS analysis indicated that 40% cells were apoptotic in p53 overexpressing HTet23p53 and HTet26p53 cells treated with OA in comparison to 8% in OA treated HeLa cells (see Additional file 5). Though, DOX treatment increases p53 transcript as well as protein levels, OA treatment does not lead to further enhancement in p53 transcript levels in HTet23p53 and HTet26p53 cells (Figure 4B and 4C). Interestingly, OA treatment significantly increases p53 protein levels (Figure 4C). Finally, ChIP assay was performed to ascertain whether in p53 overexpressing OA treated cells p53 is recruited on the promoter of its effecter genes. Results obtained indicate that indeed in p53 overexpressing cells treated with OA, p53 occupies both p21 and bax promoters (Figure 4D).

### Inhibition of Cdk5 following PP2A inhibition promotes cell survival

**Figure 5:**
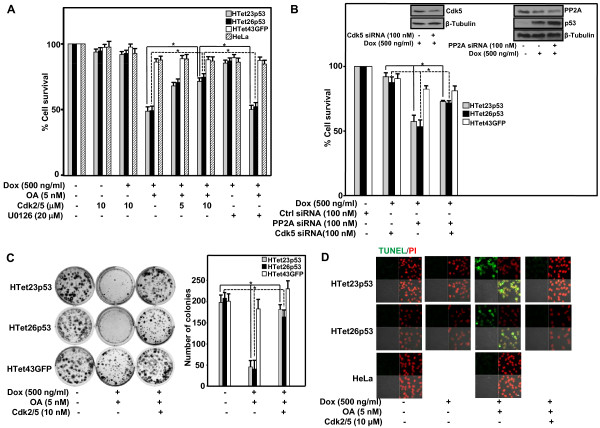
**Cdk5 inhibition rescues p53 overexpressing cells from OA induced cell-death**. (A) HTet23p53, HTet26p53, HTet43GFP and HeLa cells pretreated for 12 h with indicated concentrations of Cdk2/5 inhibitor or U0126 were treated with Dox in the presence or absence of OA and further incubated for 48 h before performing MTT assay to evaluate cell-survival. Bar graph represents variation within the wells of an experiment (± S.E.). *Represents P < 0.01. (B) HTet23p53, HTet26p53 and HTet43GFP cells plated in 96 well-plate were transfected with Ctrl or Cdk5 siRNA and incubated for 12 h. These cells were then transfected with PP2A siRNA and further incubated with Dox for 48 h. Cells were then processed for MTT assay. HTet23p53 cells were transfected with PP2A or Cdk5 siRNA and incubated with or without Dox for 48 h followed by western blotting for PP2A and p53 or Cdk5 respectively (upper panel). (C) Five hundred HTet23p53, HTet26p53 and HTet43GFP cells pretreated for 12 h with Cdk2/5 inhibitor, were treated with Dox and/or OA as mentioned in (A) and further incubated for 48 h. After 21 days cells were stained with crystal-violet and colonies were counted. Bar graph represents average colony number (± S.E.) form three independent experiments. *Represents P < 0.05. (D) HTet23p53, HTet26p53 and HeLa cells pretreated for 12 h with Cdk2/5 inhibitor followed by addition of Dox with or without OA were further incubated for 48 h and processed for apoptosis detection by TUNEL assay. Bar:10 μm.

**Figure 6:**
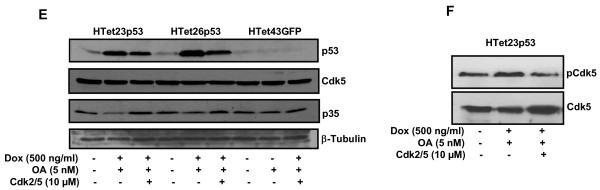
**Inhibition of Cdk5 activity reverses OA induced increase in p53 levels**. (A) HTet23p53, HTet26p53 and HTet43GFP cells pretreated for 12 h with indicated concentrations of Cdk2/5 inhibitor or U0126 were treated with Dox in the presence or absence of OA, further incubated for 48 h and processed for western blotting with p53, Cdk5 and p35 antibodies. (B) HTet23p53 cells were treated as mentioned in (A) and processed for western blot for pCdk5 (Tyr15) and Cdk5 specific antibodies.

The importance of kinases involved in the activation of p53 by OA treatment was explored by utilizing specific pharmacological inhibitors. Pre-treatment with a specific Cdk2/5 inhibitor increases cell survival whereas ERK inhibition by U0126 did not have any impact on survival (Figure 5A) of p53 overexpressing and OA treated HTet23p53 and HTet26p53 cells as compared to HTet43GFP and HeLa cells. To confirm the functional importance of PP2A and Cdk5, corresponding siRNAs were transfected into the cells. Cdk5 siRNA significantly decreased Cdk5 protein levels (Figure 4B upper left panel). Transfection with PP2A siRNA decreases its protein levels whereas p53 protein increases in addition to inhibiting cell survival (Figure 4B upper right panel). Interestingly, siRNA mediated knockdown of Cdk5 promotes survival of p53 overexpressing HTet23p53 and HTet26p53 cells as compared to HTet43GFP cells (Figure 5B). Cdk5 inhibition by its inhibitor causes significant increase in number and colony-size of p53 overexpressing HTet23p53 and HTet26p53 cells inspite of being treated with OA (Figure 5C). This result indicates that activation of p53 is dependent on the functional level of Cdk5. In p53 overexpressing cells OA treatment causes increase in apoptotic population which diminishes in the presence of Cdk5 inhibitor, as detected by TUNEL immunofluorescence staining (Figure 5D). Finally, to prove that stabilization and activation of overexpressed p53 protein is dependent on the functionality of Cdk5, cells treated with OA acid were also exposed to Cdk2/5 inhibitor. Treatment with OA increases the levels of overexpressed p53 whereas, addition of Cdk2/5 inhibitor diminishes it (Figure 6A). Neither OA nor Cdk2/5 affects the level of Cdk5 protein per se. However, the level of p35 protein decreases in the presence of OA and addition of Cdk2/5 reverts back to the basal level (Figure 6A). Finally Cdk5 activity was confirmed by increased phosphorylation level of Cdk5 tyrosine 15 residue following OA treatment (Figure 6B compare lane 2 vs lane 1) which was diminished by Cdk2/5 inhibitor (Figure 6B compare lane 3 vs lane 1).

### p53 executes apoptosis through mitochondrial pathway

**Figure 7:**
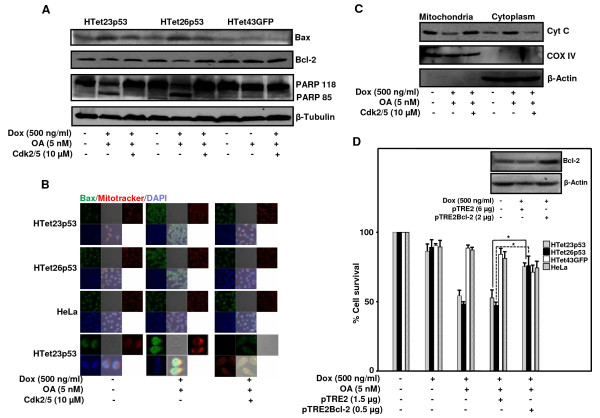
**p53-mediated apoptosis follows intrinsic mitochondrial pathway**. (A) HTet23p53, HTet26p53 and HTet43GFP cells pretreated for 12 h with Cdk2/5 inhibitor followed by Dox with or without OA were further incubated for 48 h and processed for western blotting with Bax, Bcl-2 and PARP antibodies. (B) HTet23p53, HTet26p53 and HeLa cells were treated as mentioned in (A) and incubated with mitotracker deep-red. Cells were fixed and processed for immunofluorescence with Bax antibody. Bar:10 μm. Lower panel shows magnified-view for HTet23p53 cells. (C) HTet23p53 cells were treated as described in (A) and processed for mitochondrial and cytoplasmic fractionation. Western blotting was performed with cytochrome-C antibody. (D) HTet23p53 cells transfected with pTRE or pTREBcl-2 plasmids were treated with 500 ng/ml of Dox for 48 h and processed for western blotting with Bcl-2 specific antibody. β-Actin served as a loading control (inset). HTet23p53, HTet26p53, HTet43GFP and HeLa cells were transfected with pTRE or pTRE2Bcl-2 plasmids and treated with Dox with or without OA and further incubated for 48 h. Cell-viability was determined by MTT. Bars represent variation within the wells of an experiment done twice (± S.E.). *Represents P < 0.01.

Bax and Bcl-2 levels were detected to determine the involvement of mitochondrial pathway. Though Bax was upregulated following OA treatment, its upregulated status was reverted back in the presence of Cdk2/5 inhibitor in HTet23p53 and HTet26p53 cells overexpressing p53 (Figure 7A). Complementary to Bax upregulation, Bcl-2, which heterodimerizes and interferes with Bax homodimerization, was downregulated and its level was normalized back to basal expression in the presence of Cdk2/5 inhibitor, in p53 overexpressing OA treated cells (Figure 7A). No alterations in Bax and Bcl-2 were observed in HTet43GFP cells. Further, to confirm that Cdk2/5 inhibitor actually inhibits apoptosis; PARP was detected by western blot. Cleavage of PARP into p85 peptide was detected only in p53 overexpressing HTet23p53 and HTet26p53 cells (Figure 7A). Immunofluorescence studies revealed increased mitochondrial localization of Bax in p53 overexpressing OA treated cells, which was diminished by Cdk2/5 inhibitor (Figure 7B). HeLa cells served as control for these studies. In p53 overexpressing OA treated cells decreased mitochondrial cytochrome-C (Cyt-C) and increased cytosolic levels were observed (Figure 7C). Finally, to ascertain the mitochondrial apoptosis, Bcl-2, was ectopically expressed (Figure 7D inset). As expected significant decreased apoptotic cells were detected in p53 overexpressing OA treated HTet23p53 and HTet26p53 cells in comparison to vector alone transfected or in HTet43GFP and HeLa cells (Figure 7D).

### Cdk5 interacts to phosphorylate p53

**Figure 8:**
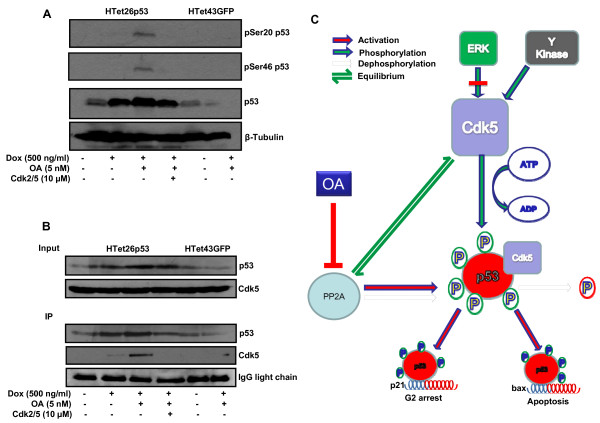
**Cdk5 associates with p53 to phosphorylate at Ser20 and Ser46**. (A) HTet26p53 and HTet43GFP cells 12 h pretreated with Cdk2/5 inhibitor were treated with Dox in the presence or absence of OA for 48 h and processed for western blotting with p53, pSer20 and pSer46 antibodies. (B) HTet26p53 and HTet43GFP cells treated as mentioned in (A) were processed for immunoprecipitation with p53 antibody. p53 and Cdk5 immunoblot was done. (C) Model for Cdk5-mediated p53 phosphorylation and activation. Cdk5 phosphorylates p53 which is dephosphorylated by PP2A. Inhibition of PP2A promotes phosphorylation of p53 at Ser20 and Ser46. Activated p53 is recruited on p21 and Bax promoter to execute cell cycle arrest and apoptosis. Cdk5 phosphorylation is not dependent on ERK activation but on an unknown kinase(s).

Under genotoxic stress conditions activation of p53 is achieved by phosphorylation at Ser20 and Ser46 residues [26,27]. To explore that in p53 overexpressing OA treated cells Cdk5 plays an important role, phosphorylation status of Ser20 and Ser46 was detected in the presence or absence of OA. Phosphorylation at Ser20 and Ser46 residues of overexpressed p53 increased significantly in OA treated cells, whereas in the presence of Cdk2/5 inhibitor phosphorylated forms diminished (Figure 8A). Under identical experimental conditions no increased phosphorylation was detected in HTet43GFP cells. Finally, to ascertain whether Cdk5 associates with p53 to cause its phosphorylation, co-immunoprecipitation experiment was performed by immunoprecipitating p53 with its specific antibodies and this immuno-complex was probed with Cdk5 antibody by western blotting as described in materials and methods section. Interestingly, Cdk5 was detected in immuno-complex isolated from p53 overexpressing OA treated HTet26p53 cells. In the presence of Cdk2/5 inhibitor this interaction was reduced (Figure 8B).

### Activated and not overexpressed p53 inhibits tumor growth

**Figure 9:**
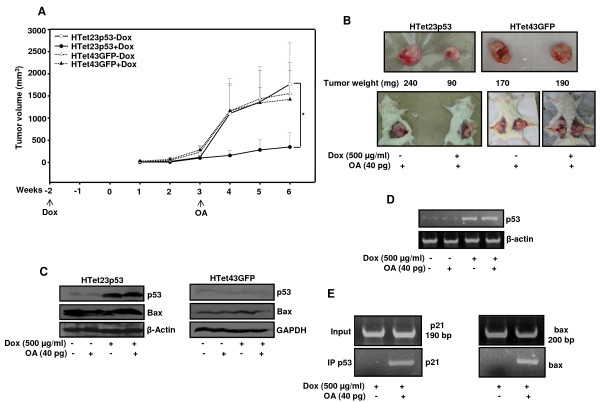
**Activated p53 inhibits tumor-growth by transcriptional activation of its downstream pathways**. (A) HTet23p53 (n = 12 mice) or HTet43GFP (n = 4 mice bearing 2 tumors each) were divided in two groups and one group was given water supplemented with Dox. After tumor size reached to 5-10 mm in diameter, OA was administered to all mice. Tumor-growth was measured weekly and average tumor volume was plotted (+S.E.). *Represents P < 0.05. (B) Tumor image and weight after mice were sacrificed. (C) Western blotting of p53 and bax and (D) RT-PCR for p53 was performed in HTet23p53 and HTet43GFP tumors samples from mice with or without access to Dox in the presence and absence of OA. β-actin loading control. (E) Tumors samples from mice given Dox with or without OA treatment were processed for ChIP assay. Input and eluted DNA was used for RT-PCR with p21 or bax promoter primers.

To validate that these *in vitro *findings have *in vivo *implications also, HTet23p53 or HTet43GFP cells were administered in NOD/SCID mice and monitored weekly for tumor growth. Up to three weeks after implanting cells tumors grew identically in mice supplemented with or without Dox. Thereafter, tumor growth was rapid in mice injected with HTet23p53 cells and treated with OA without being supplemented with Dox. Similarly, in mice injected with HTet43GFP cells, tumors grew rapidly in those treated with OA and supplemented with or without Dox. Interestingly, in mice injected with HTet23p53 cells and treated with OA,in addition to being supplemented with Dox, tumor growth was significantly retarded (Figure 9A). Reduced tumor-growth is reflected in differences in size and weight of the excised tumors (Figure 9B). Tumor samples were analyzed to ascertain the involvement of stabilized p53 and also for the activation of its downstream growth inhibitory factors. In tumor samples from OA treated mice bearing HTet23p53 cells, p53 and bax protein levels were higher (Figure 9C) and these did not increase in tumors of HTet43GFP cells. p53 transcript and protein levels were higher in HTet23p53 cells derived tumors from mice supplemented with Dox and, levels were not enhanced further by OA treatment (Figure 9D). These results clearly indicate that the stabilization of p53 protein also occurs in *in vivo *tumors. Conclusively, the ChIP assays performed on lysates of tumors excised from mice provided with Dox in water, with or without OA treatment revealed enhanced promoter occupancy of activated p53 on p21 and bax promoters *in vivo *(Figure 9E).

## Discussion

This study highlights the activation of overexpressed p53 and its effect on cell cycle arrest and apoptosis in HPV-positive HeLa cells. Under stress conditions p53 is stabilized by phosphorylation and acetylation at serine/threonine/tyrosine and lysine residues respectively. The serine phosphorylation at residues 6,9,15,20,33,37,46,315 and 392 plays a crucial role depending upon the nature of stress thereby causing cell cycle arrest and/or apoptosis [26,27]. Unlike stress condition wherein p53 induction promotes cell cycle arrest or apoptosis, this study demonstrates that p53 overexpression in HPV-positive cells does not induce cell cycle arrest or apoptosis though; it is reported to do so in other cancer cell types [16,17,28]. The reason for this difference could be inhibition of cellular machinery necessary for performing critical posttranslational modifications which are required for sequence specific promoter selection of the genes responsible for the induction of cell cycle arrest or apoptosis by HPV [5,29].

Equilibrium between phosphorylation and dephosphorylation of a protein like p53 is essential for its normal functioning in the cells. Therefore, conditions causing shift in the equilibrium between phosphorylated and non-phosphorylated states will dictate the functionality of a protein and subsequently the cells fate [30,31]. Protein phosphatases inactivate p53 by dephosphorylating it. Very recently Lu *et al*., reported that PP2A inhibition also decreases p53 protein and its phosphorylation at Ser15 through activation of its negative regulator MDM2 [32]. In contrary, we herein demonstrate that inhibition of phosphatase stabilizes and activates overexpressed p53 probably because of impairment in functional MDM2 pathway in HPV-positive cells [33]. Phosphorylation of p53 at specific serine residues is essential for the induction of cell cycle arrest and apoptosis. Under stress conditions p53 is phosphorylated at Ser20 located in the transactivation domain [26], thereby stabilizing and triggering downstream pathways. Ser46 phosphorylation, located in the DNA-binding domain of p53 plays a crucial role in sequence specific DNA-binding required for the induction of cell cycle arrest and apoptosis [34]. In this study, we confirm that phosphorylation at these residues fully restores p53 functionality and induces cell-death even under non-stress conditions.

Stress-induced p53 is stabilized and activated by various kinases such as ATM, ATR, Chk1, HIPK2 and Chk2 by phosphorylation [26,27,34,35]. However, very little is known about the kinases that phosphorylate p53 under non-stressed conditions. Cdk5 was originally discovered in HeLa cells [36] and its functional role as p53 upstream kinase has been documented in neuronal cells [37]. Involvement of Cdk5 in growth of breast and prostate cancers cells has been reported [38-40]. Recently, we reported that Cdk5 transactivates p53 in breast cancer cells under positive regulation of ERK following carboplatin treatment [40]. Cdk5-inhibition promotes survival of p53 expressing cells. As PP2A-inhibition restores the ability of overexpressed p53 to promote cell-death, the upstream kinase that phosphorylates overexpressed p53 under non-stress conditions was investigated. In the present study we demonstrate that p35, a Cdk5 activator levels diminishes following inhibition of PP2A and simultaneous increase in the levels of more sustainable Cdk5 activator p25 following p35 cleavage [41]. Thus, increased level of Cdk5 activator (p25) may facilitate Cdk5-mediated phosphorylation of overexpressed p53, which causes cell-growth inhibition. The decreased level of p35 protein in HTet43GFP cells does not cause cell-growth inhibition because of unavailability of its substrate (in this model overexpressed p53). Though, Cdk5 plays an important role in activating overexpressed p53, as such it is not involved in the proliferation of parental HeLa cells *per se *in spite of the fact that E6 expression leads to increase in Cdk5 protein expression.

p53 executes its apoptotic function through intrinsic or extrinsic pathways [42,43]. To further confirm the pathway involved, we investigated Bax, an important transcriptional target of p53 involved in promoting intrinsic mitochondrial apoptosis. Bax translocates to mitochondrial outer membrane causing MOMP and releases cytochrome-C into cytosol. Cells lacking Bax or those overexpressing Bcl-2 are profoundly resistant to a broad range of apoptotic stimuli, including chemotherapeutic drugs treatment and serum starvation [17]. In HPV-positive cancers Bcl-2 overexpression and Bax degradation by E6 facilitates cancer progression [14]. Here, we demonstrate that upregulated Bax translocates to mitochondria upon PP2A-inhibition in p53 overexpressing cells which is dependent on Cdk5 activity. Thus, only phosphorylated p53 triggers Bax transcription to increase its levels and cause apoptosis. In addition, the cell cycle arrest caused by inhibition of PP2A in p53 overexpressing cells may be dependent on transcriptional upregulation of p21 gene. Collectively these data also provide evidence for reactivation of E6 disrupted p21 and Bax pathways in HPV positive cells.

Finally, we propose that Cdk5 interacts with p53 and phosphorylates Ser20 and Ser46 residues. Phosphorylation restores the ability of overexpressed p53 to specifically bind on p21 and bax promoters (Figure 5*C*). These findings provide novel insight into the regulation of p53 transactivation functions and propose PP2A to be a key player in modulating p53 functionality. The phosphorylated status of specific residues may be involved in promoter selection and this proposition needs further investigations. Also, this is the first report which provides mechanism for functional activation of p53, and details the essential modifications necessary for non-genotoxically overexpressed p53 to be able to execute its tumor suppressor functions in HPV-positive cells. Moreover, activation of overexpressed p53 without targeting viral oncogenes may have implication in the treatment of virus infected carcinomas. The efforts towards the newer approaches to target p53 pathway and usefulness of reactivation of p53 pathways in treatment of cancers are encouraging. Therefore, these findings could have therapeutic importance for the treatment of cervical cancers as well as other cancers types in which p53 is functionally abrogated.

## Abbreviations

CAT: chloramphenicol acetyl transferase; CDK5: cyclin dependent kinase 5; DOX: doxycycline; OA: okadaic acid; PP2A: protein phosphatase 2A;

## Competing interests

The authors declare that they have no competing interests.

## Authors' contributions

AKA performed most of the experiments and prepared the manuscript. AKU and SS helped in manuscript preparation. MVV repeated animal experiment. RK and VP helped in tumor weight and excision. BR helped in mice daily maintenance. MKB conceived the study, participated in its design and coordination, corrected the manuscript and supervised the project. All authors read and approved the final manuscript.

## Supplementary Material

Additional file 1 **Material and methods**. File contains material, methods and references pertaining to additional files 2, 3, 4, 5. Click here for file

Additional file 2 **Verification and screening for Tet-On responsive p53/GFP clones**. (A) For screening of Tet-On as well as pTREp53 transfected cells, clones (HeLa-Tet-On-p53 numbered 21 to 44) were treated with 2000 ng/ml Dox for 48 h and processed for western blotting with p53 specific antibody. β-Actin served as a loading control. Two clones with low leaky and high inducible expression, HeLa-Tet-On-p53 23 and 26, were selected for further studies. (B) Screening for Tet-On regulated GFP clones (HeLa-Tet-On-pBIGFP number 41 to 52) was performed by incubating cells with 2000 ng/ml Dox for 48 h followed by observation under fluorescent microscope. One clone HeLa-Tet-On-pBIEGFP (number 43) having low leaky and high inducible expression was selected for further studies. Click here for file

Additional file 3 **Growth properties of selected clones did not alter in comparison to parental HeLa cells and Dox upto 2000 ng/ml is not significantly toxic to cells**. (A) To evaluate viable cells over a period of 6 days, 2000 cells plated in triplicate in a 96 well plate were stained with trypan blue. Graphs were plotted with viable cell number *vs *growth days. (B) Cells were plated as mentioned in *A *and lysed for protein estimation. Graph was plotted with protein concentration *vs *days. (C) Ten thousand cells plated in triplicate in a 96 well plate were treated with indicated concentration of Dox for 48 h and processed for MTT assay. Graph was plotted with percentage cell survival *vs *Dox concentration. Click here for file

Additional file 4 **Overexpressed p53 exhibits nuclear localization**. (A) HTet23p53, (B) HTet26p53 and (D) HeLa cells treated with 100 and 1000 ng/ml of Dox were incubated for 48 h and processed for immunofluorescence study with p53 specific antibody. HTet23p53, HTet26p53 and HeLa cells were probed with FITC conjugated (green-staining). Upper left section of image represents p53 staining (green); lower left represents DAPI (blue), upper right phase contrast and lower right (overlay of them). (C) HTet43GFP cells were probed with rhodamine conjugated (red-staining) 2° antibody. Green staining in HTet43GFP cells depicts GFP staining. DAPI (blue staining) represents nuclear staining. Upper left section of image represents GFP (green), upper middle section represents p53 staining (red), lower left represents DAPI (blue), upper right phase contrast and lower middle represents (overlay of them). Bar:10 μm. Click here for file

Additional file 5 **Activated p53 triggers apoptosis**. HTet23p53, HTet26p53 and HeLa cells were plated in a 35 mm plate and pretreated with Cdk2/5 (10 μM) inhibitor for 12 h. Thereafter 500 ng/ml Dox or 5 nM OA was added for 48 h. Cells were harvested by trypsinization and processed for TUNEL assay by FACS analysis as per manufacturer's instruction with the modification that reaction mixture was incubated for 2 h instead of 1 h. Click here for file
