# Supplementary material for: Cdk5 phosphorylates non-genotoxically overexpressed p53 following inhibition of PP2A to induce cell cycle arrest/apoptosis and inhibits tumor progression
Source: Mol Cancer. 2010 Jul 31;9:204. doi: 10.1186/1476-4598-9-204 (PMC2922192; doi:10.1186/1476-4598-9-204)
Supplement: Additional file 1 — Material and methods. File contains material, methods and references pertaining to additional files 2, 3, 4, 5. [file 1476-4598-9-204-S1.PDF]

Materials and methods

Cell-lines, transfection and development of p53 inducible Tet-On isogenic system. HeLa cell-line was purchased from American Type Culture Collection (Manassas, VA) and maintained in our in-house National Cell Repository, National Centre for Cell Science (NCCS), Pune, India. All the cell-lines were regularly cultured in DMEM supplemented with 10% heat inactivated FBS (Tet system approved FBS for inducible cell-lines and standard FBS for HeLa cells) at 37^0C with 5% CO_2. Inducible cell-lines were maintained in 100 µg/ml of G418 and 50 µg/ml of hygromycin B whereas HeLa cells were grown in 100 U/ml of penicillin and 100 µg/ml streptomycin. pTet-On, pTRE, pTRE2, pTK-Hyg and pBIEGFP were purchased from Clontech.

HeLa cells were transfected with pTet-On and selected on G418 for 21 days with the replacement of fresh media containing same concentration of G418 every 4 day to develop HeLaTet-On cells. HeLaTet-On cells were verified by transfecting pBIEGFP and observing GFP fluorescence after 48 h induction with 2000 ng/ml of Dox. Clones expressing high GFP and low leaky expression were selected for the development of double stable cell-line. HeLaTet-On cells were cotransfected with pTREp53 or pBIEGFP and pTKHyg. Cells were selected for 21 days on 200 µg/ml of hygromycin B. Total 24 colonies were picked up and screened for p53 expression after addition of 2000 ng/ml of Dox for 48 h. For GFP 12 colonies were picked up and after 2000 ng/ml Dox addition cells were observed under fluorescent microscope.

Western blotting. Western blot analysis was performed with specific antibodies as per the protocol described earlier (1). Where ever possible blots were stripped by incubating the membranes at 50^0C for 30 min in stripping buffer (62.5 mM Tris-Cl pH 6.7, 100 mM mercaptoethanol, 2% SDS) with intermittent shaking. Membranes were washed thoroughly with TBS and reprobed with required antibodies. Otherwise gels run in duplicates were probed for the desired proteins by western blotting and then compiled.

Cell-viability. Cell-viability assay was performed by trypan blue dye exclusion method. After treatment with the indicated concentrations of Dox cells were incubated at room temperature with 0.1% trypan blue in PBS for 5 min. Trypan blue positive as well as total cell population was counted to calculate the percent viable cells.

TUNEL Assay. APO-DIRECT TUNEL assay kit (BD) was used to detect apoptotic cell population by flow cytometric analysis or confocal as per manufacturer's instructions with some modifications. Cells were incubated in DNA-labeling solution for 2 h at 37^0C and analyzed by FACS Calibur (BD). PI stains total DNA and FITC conjugated dUTP stains apoptotic cells.

Immunofluorescence study. Cells grown on Labtek chamber slides followed by Dox treatment for 48 h were processed for immunofluorescene study as described earlier (2). Primary antibody against p53 (1:50) was added and incubated for 2 h at room temperature. Following incubation, cells were washed 5 times. Fluorescein isothiocyanate (FITC) or Rhodamine conjugated secondary antibodies (1:100) were

added and incubated for 1 h at room temperature. After five washes, vectashield mounting medium containing DAPI was added before slides were examined on a confocal microscope (LSM510, Carl Zeiss, Germany).

References.

1. Upadhyay, A.K., Ajay, A.K., Singh, S., and Bhat, M.K. (2008). Cell cycle regulatory protein 5 (Cdk5) is a novel downstream target of ERK in carboplatin induced death of breast cancer cells. Curr. Cancer Drug Targets. *8*, 741-752.

2. Chhipa, R.R., Kumari, R., Upadhyay, A.K., and Bhat, M.K. (2007). Abrogation of p53 by its antisense in MCF-7 breast carcinoma cells increases cyclin D1 via activation of Akt and promotion of cell proliferation. Exp. Cell Res. *313*, 3945-3958.
