# Supplementary figures and images for: Cdk5 phosphorylates non-genotoxically overexpressed p53 following inhibition of PP2A to induce cell cycle arrest/apoptosis and inhibits tumor progression
Source: Mol Cancer. 2010 Jul 31;9:204. doi: 10.1186/1476-4598-9-204 (PMC2922192; doi:10.1186/1476-4598-9-204)

**A**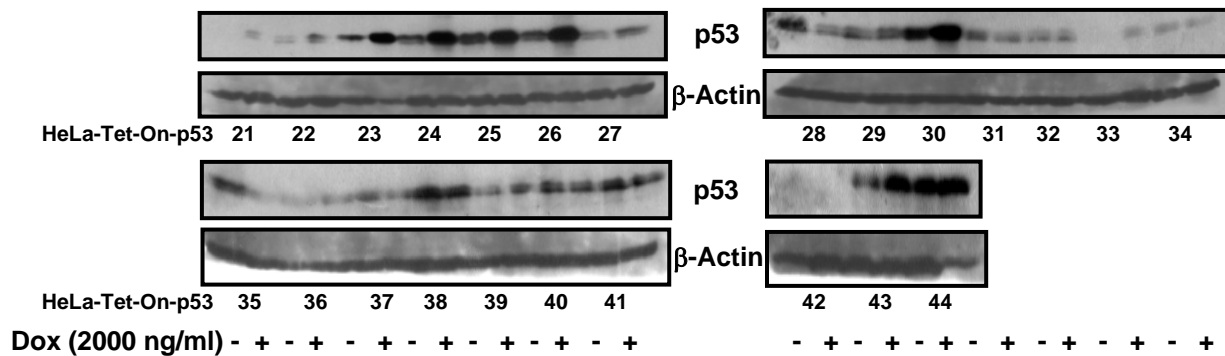**B**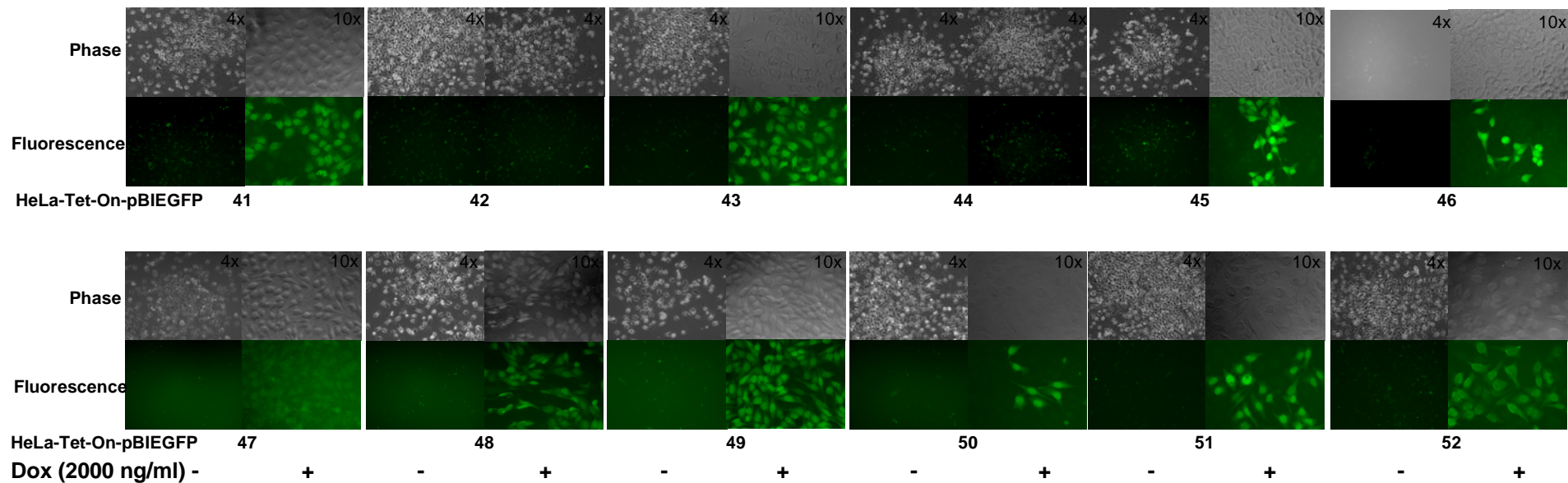

Supplement: Additional file 2 — Verification and screening for Tet-On responsive p53/GFP clones. (A) For screening of Tet-On as well as pTREp53 transfected cells, clones (HeLa-Tet-On-p53 numbered 21 to 44) were treated with 2000 ng/ml Dox for 48 h and processed for western blotting with p53 specific antibody. β-Actin served as a loading control. Two clones with low leaky and high inducible expression, HeLa-Tet-On-p53 23 and 26, were selected for further studies. (B) Screening for Tet-On regulated GFP clones (HeLa-Tet-On-pBIGFP number 41 to 52) was performed by incubating cells with 2000 ng/ml Dox for 48 h followed by observation under fluorescent microscope. One clone HeLa-Tet-On-pBIEGFP (number 43) having low leaky and high inducible expression was selected for further studies. [file 1476-4598-9-204-S2.PDF]

**A**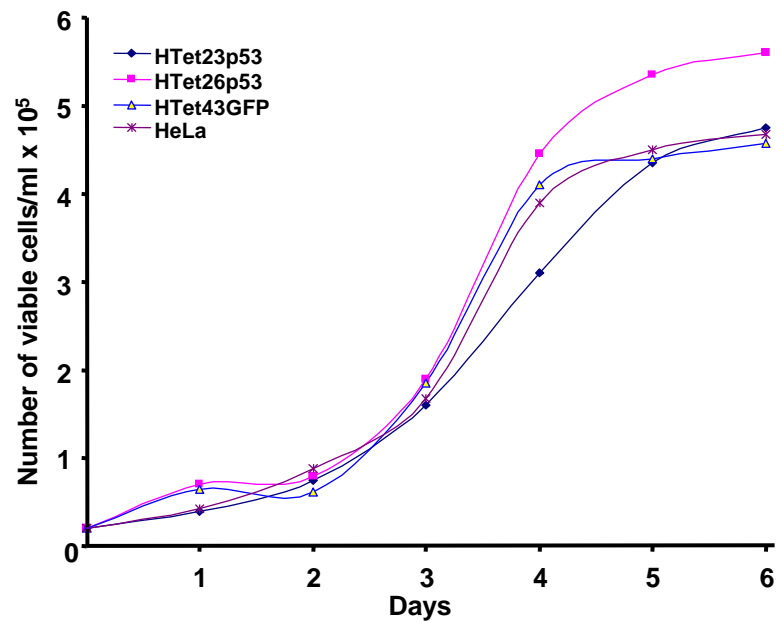**B**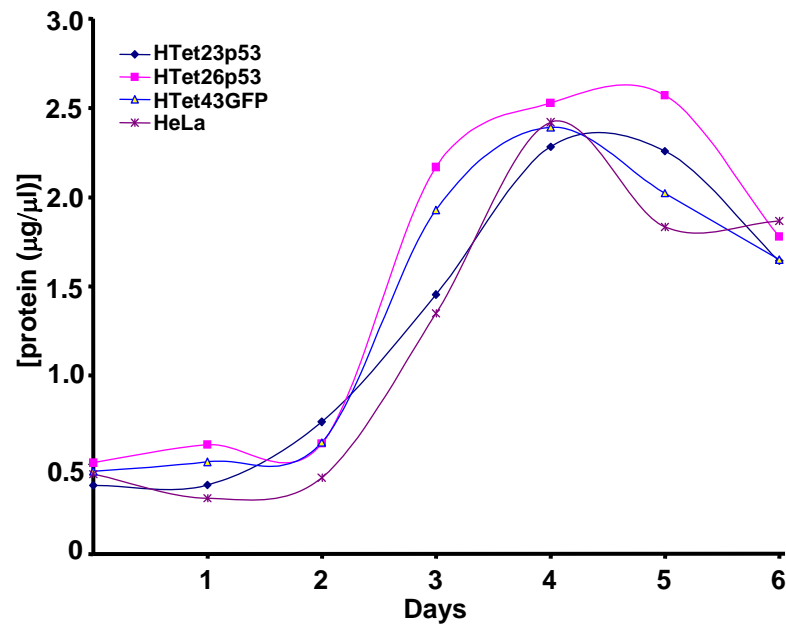**C**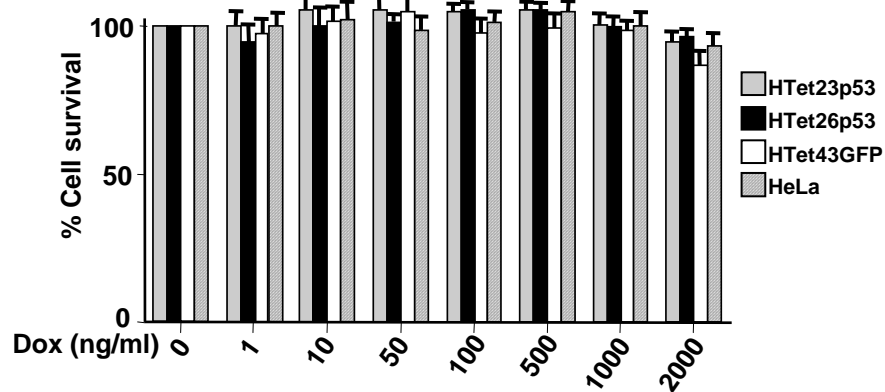

Supplement: Additional file 3 — Growth properties of selected clones did not alter in comparison to parental HeLa cells and Dox upto 2000 ng/ml is not significantly toxic to cells. (A) To evaluate viable cells over a period of 6 days, 2000 cells plated in triplicate in a 96 well plate were stained with trypan blue. Graphs were plotted with viable cell number vs growth days. (B) Cells were plated as mentioned in A and lysed for protein estimation. Graph was plotted with protein concentration vs days. (C) Ten thousand cells plated in triplicate in a 96 well plate were treated with indicated concentration of Dox for 48 h and processed for MTT assay. Graph was plotted with percentage cell survival vs Dox concentration. [file 1476-4598-9-204-S3.PDF]

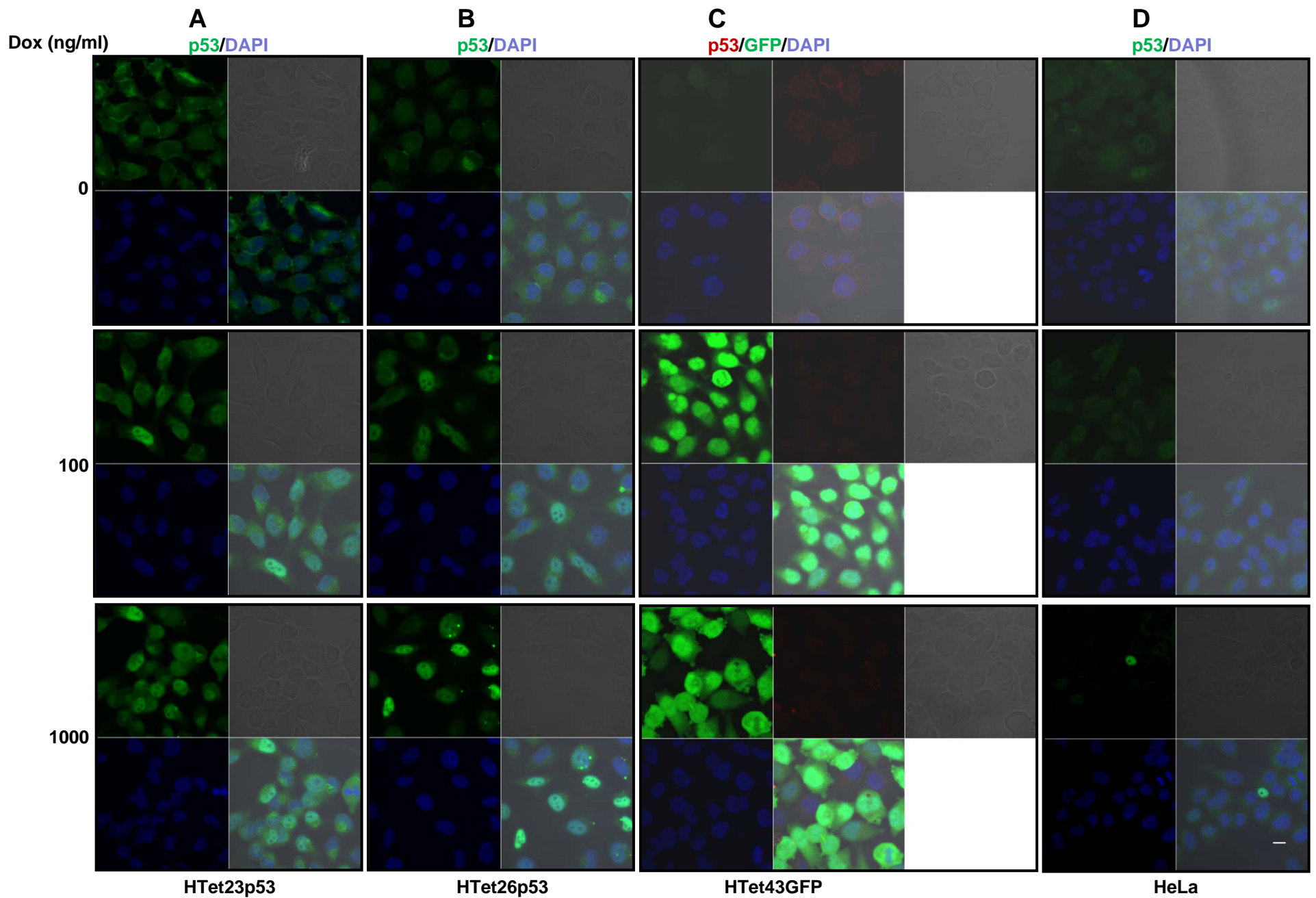

*Additional Figure 4 Ajay et. al., 2010*

Supplement: Additional file 4 — Overexpressed p53 exhibits nuclear localization. (A) HTet23p53, (B) HTet26p53 and (D) HeLa cells treated with 100 and 1000 ng/ml of Dox were incubated for 48 h and processed for immunofluorescence study with p53 specific antibody. HTet23p53, HTet26p53 and HeLa cells were probed with FITC conjugated (green-staining). Upper left section of image represents p53 staining (green); lower left represents DAPI (blue), upper right phase contrast and lower right (overlay of them). (C) HTet43GFP cells were probed with rhodamine conjugated (red-staining) 2° antibody. Green staining in HTet43GFP cells depicts GFP staining. DAPI (blue staining) represents nuclear staining. Upper left section of image represents GFP (green), upper middle section represents p53 staining (red), lower left represents DAPI (blue), upper right phase contrast and lower middle represents (overlay of them). Bar:10 μm. [file 1476-4598-9-204-S4.PDF]

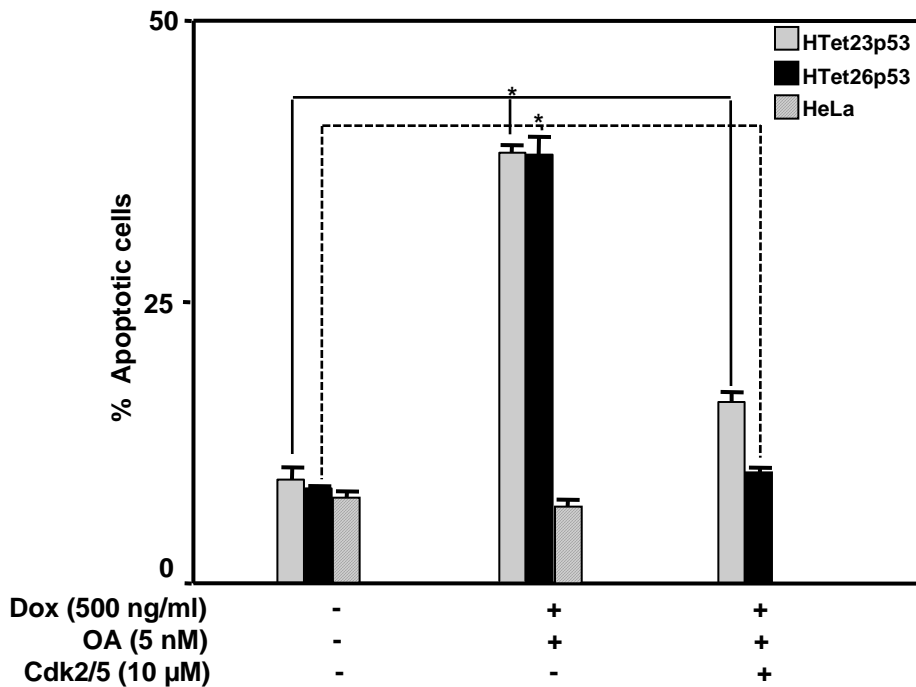

*Additional Figure 5 Ajay et. al., 2010*

Supplement: Additional file 5 — Activated p53 triggers apoptosis. HTet23p53, HTet26p53 and HeLa cells were plated in a 35 mm plate and pretreated with Cdk2/5 (10 μM) inhibitor for 12 h. Thereafter 500 ng/ml Dox or 5 nM OA was added for 48 h. Cells were harvested by trypsinization and processed for TUNEL assay by FACS analysis as per manufacturer's instruction with the modification that reaction mixture was incubated for 2 h instead of 1 h. [file 1476-4598-9-204-S5.PDF]
